# Comparison of Standard and Intensified Regimens for HIV-Negative Adults With Tuberculous Meningitis in West China: A Retrospective Observational Study

**DOI:** 10.3389/fneur.2019.00626

**Published:** 2019-06-13

**Authors:** Ammar Taha Abdullah Abdulaziz, Yi Meng Ren, Wei Li, Jin Mei Li, Dong Zhou

**Affiliations:** ^1^Neurology Department, West China Hospital of Sichuan University, Chengdu, China; ^2^Queen Mary School, Nanchang University, Nanchang, China

**Keywords:** tuberculous meningitis, standard therapy, levofloxacin, moxifloxacin, hydrocephalus

## Abstract

**Background:** Tuberculous meningitis (TBM) is an extremely devastating inflammation of the central nervous system; however, no available optimum treatment can effectively control the disease so far.

**Method:** The medical records of TBM patients from May 2011 to August 2016 in West China hospital were retrospectively analyzed. Patients were divided into three groups based on their treatment regimens {Group1: 4 standard therapy; Group2: 3 standard drugs + Levofloxacin; Group3: 4 standard therapy + Levofloxacin (G3a)/ Moxifloxacin (G3b)}. Using the intention-to-treat analysis, eventually, the treatments' efficacy and safety were compared among all groups.

**Results:** Two hundred two patients with TBM were enrolled and followed up for at least 2 years. Among them, 99 patients were in G1; 18 in G2; and 85 in G3 (Moxifloxacin=39/ Levofloxacin=49). One hundred fifteen (56.9%) patients were males, and the median age was 42 years. At admission, 74 patients (36.6%) were in stage I, 102 (50.5%) in stage II and 26 (12.9%) in stage III. The most common symptoms were headache in 194 (96.0%) patients, fever in 162 (80.2%), vomiting in 120 (59.7%), neck stiffness in 104 (51.5%), and malaise in 96 (47.5%). The overall outcome at 1 year showed that 47 patients (47.5%) in G1, 10 patients (55.6%) in G2 and 48 patients (56.5%) in G3 had good outcome; however, there was no significant difference among all groups (*P* = 0.397); at 2 years there was also no difference among treatment groups (*P* = 0.295). However, in Group3b 22 patients (56.4%) at 1-year and 26 (66.7%) at 2-year follow up had a full recovery, which is significantly superior to other treatment groups, the *P* value at 1 and 2 years was 0.002 and 0.027, respectively.

**Conclusion:** The overall outcome in patients with TBM at 1 and 2 years follow up did not show any statistically significant difference between the standard chemotherapy and other intensified regimens. Furthermore, Hydrocephalus (OR = 3.461, 95% CI: 1.349–8.882, *P* = 0.010) was the only independent risk factor for a poor outcome.

## Background

Tuberculosis (TB) remains the 9th leading cause of death globally, ranking above HIV/AIDS, and difficulties in tuberculosis diagnosis have delayed progress in reducing the burden of disease, especially in endemic areas (1). In 2016, the World Health Organization (WHO) estimated that 10.4 million people fell ill with TB and 56% were found in five developing countries including China (1). Tuberculous meningitis (TBM), the most devastating form of tuberculosis infection, results in death, or neurological disability in up to 50% of affected patients (2, 3).

The diagnosis of TBM usually relies on clinical evidence, which combines supportive clinical, laboratory, and radiological findings. For decades, the absence of standardized diagnostic guidelines made the comparison of research findings difficult, prevented the best use of the existing data, and limited progress in management. However, the recently published diagnostic criteria by Marais et al. (4) has been widely adopted and become the most commonly used diagnostic guidelines for TBM (4).

The current WHO guidelines for TBM treatment recommend the administration of four anti-tuberculosis drugs (Isoniazid, Rifampicin, Pyrazinamide, and Ethambutol) for at least the first 2 months of therapy after that treatment continued with two drugs (rifampicin and isoniazid) for an additional 7–10 months (5). However, these guidelines are based on data from pulmonary TB and do not take into account the limited ability of anti-TB medications to penetrate the brain.

Fluoroquinolones are active anti-tuberculosis agents with good penetration into the blood-brain Barrier (6). These medications may improve the outcome of TBM by enhancing the initial anti-mycobacterial activity of the standard anti-tuberculosis drug regimen. Additionally, they may be useful alternatives for patients unable to take isoniazid or rifampicin due to either patient's intolerance or bacterial drug resistance to these agents. There are few recently published data suggesting the use of one of the fluoroquinolones, particularly either levofloxacin or moxifloxacin for TBM treatment (6–9), however, the optimal fluoroquinolone agent and the exposure for TBM treatment have not been defined yet. Moxifloxacin among fluoroquinolones has the highest activity against *Mycobacterium tuberculosis in vitro* and mice (7, 10). Thereby, it could be an essential addition in the treatment of TBM (11). A randomized study involving Vietnamese adults with tuberculous meningitis suggested that the initial addition of levofloxacin to a standard four-drug anti-tuberculosis regimen improved the survival rate, especially among patients who were treated before the onset of coma (6). However, a recent clinical trial reported that the intensified regimen with levofloxacin was not associated with improved survival compared to the standard therapy over 9 months of follow-up with 28% mortality in both arms (12).

To date, the optimal treatment regimen for TBM is still unclear. Therefore, this study aimed to compare the efficacy, outcome, and complications of different intensified regimens for TBM, which may provide an essential clue forward the setting of the optimum anti-TB regimen.

## Methods

### Study Design

The medical records of all patients older than 16 years with tuberculous meningitis, who were admitted to West China Hospital of Sichuan University (the biggest tertiary care hospital in west China) from May 2011 to August 2016 were retrospectively reviewed. Patients who met the recommended diagnostic criteria published by Marais et al. (4) were eligible for this study (3). Exclusion criteria were: Incomplete medical records; interrupted or changed treatment; Failure to do a diagnostic lumbar puncture; Evidence of bacterial/fungal Meningitis; Pregnancy/ lactation; Known contraindication for drugs / hypersensitivity / intolerance; Sexually transmitted diseases; Known or suspected multi-drug resistance; Previous administration of TB medications. All enrolled patients underwent neuroimaging studies and chest CT at baseline and divided into subgroups based on their treatment regimens. Eventually, the clinical characteristics, laboratory and cerebrospinal fluid (CSF) findings, radiological abnormalities and outcomes were compared and contrasted among the different groups.

### Data Collection

Demographic data, clinical manifestations, laboratory results, radiological findings, treatment, and the duration from disease onset to admission were collected. In addition, hospitalization time, follow-up times were documented. The clinical severity at admission was staged according to the British Medical Research Council (BMC) criteria for TBM (13). Grade 1 corresponds to alert and oriented patient without focal neurological deficits. Grade 2 is defined as a Glasgow coma scale of 11 to 14 or 15 with minor focal neurological deficits. Grade 3 is defined as a Glasgow coma scale of 10 or less with or without focal neurological deficits. The overall outcomes of all groups were evaluated and compared, respectively.

### Treatment

The study population was divided into three major groups based on their treatment regimens (drugs' number and type):

^*^G1 (4st.): standard therapy included: Isoniazid, Rifampicin, Ethambutol, and Pyrazinamide.
^*^G2 (3st.+L): intensified 4 drugs therapy included: Isoniazid, Rifampicin, Ethambutol, and Levofloxacin.
^*^G3a (4st.+L): intensified 5 drugs therapy included: 4 standard therapy plus Levofloxacin.
^*^G3b (4st.+M): intensified 5 drugs therapy included: 4 standard therapy plus Moxifloxacin.

All patients in G1 and G3 received the 4 anti-TB standard chemotherapy (Isoniazid 5 mg/kg/day; Rifampicin 10 mg/kg/day; Pyrazinamide 25 mg/kg/day; and Ethambutol 20 mg/kg/day) for the first 3 months, thereafter treatment continued with only Isoniazid, and Rifampicin for at least 9 months. In addition, fluoroquinolones (Moxifloxacin 400 mg or Levofloxacin 20 mg/kg/day) were administrated in the first 2 months on a daily basis for G3 patients. While the patients in G2 had received only 3 out of the 4 standard anti-TB drugs (Isoniazid, Rifampicin, and Ethambutol) for the first 3 months and Levofloxacin for just the first 2 months. Later on, they received the same treatment as G1 and G3. Rifampicin and Levofloxacin were given intravenously to the intensified groups during the hospitalization period; other drugs were given orally, while unconscious patients received their medication through the nasogastric tube. Moreover, all participants received adjunctive intravenous dexamethasone (0.4 mg/kg/24 h), and the dose was tapered over 8 weeks as recommended (14).

### Follow-Up and Assessment of Outcome

Patients were followed up to 4 years (mean = 2.5 years) after the initiation of treatment. The daily records of patients' condition until discharge from the hospital were reviewed for assessment of clinical progress and neurologic, drug-related adverse events and other clinical parameters. After discharge, patients were followed up at different time points in our center or local centers for clinical evaluation and laboratory monitoring, and later by calling to assess the long term outcomes and complications. The primary outcome was the full recovery rate in each group at 12, 18, and 24 months after the initiation of treatment. The secondary outcome included drug-related adverse events in different study groups and the impact of hospitalization delay, neurologic events, biochemical, and radiological findings on the overall long term outcome. Patients were at least evaluated through follow up visits at 3, 6, and 12 months, or up to 2 years if they were not fully recovered at 12 months, and then by phone calls. The outcome was also assessed using the modified Rankin Scale (mRS) at 12 and 24 months. Patients were considered fully recovered if they have no symptoms (mRS 0); intermediate if they were independent in activities of daily living despite some symptoms or slight disability (mRS 1–2); severely disabled for all other cases except death (mRS 3–5); or dead (mRS 6) (14, 15). All patients were distributed into 2 groups: those with a complete or intermediate recovery (mRS 0–2) were considered as having a good outcome; while patients with severe symptoms, disability, or death (mRS 3–6) were considered as having a poor outcome. Full recovery was defined as complete remission of TBM symptoms without any degree of disability. While a neurologic event was defined as the occurrence of any of the following: cranial nerve palsy; cerebellar symptoms; monoplegia, hemiplegia, paraplegia, or tetraplegia; seizures; or a decrease in Glasgow coma score of 2 or more points during hospitalization. Moreover, drug-induced hepatotoxicity was defined as ALT/ AST > 2 times upper limit of the normal range; Hematotoxicity was defined as the occurrence of anemia (Hb ≤ 80 ×10^12^/L), leukopenia ( ≤ 2.0 ×10^9^/L) or thrombocytopenia (<100 ×10^9^/L), whereas Cardiotoxicity was defined as the development of arrhythmia.

### Statistical Analysis

All statistical analyses were performed using SPSS software (version 21.0). Data were expressed as frequency along with percentage and mean (±SD) with median and range. Descriptive statistics were used to summarize demographic and all other clinical characteristics of the patients. One-way ANOVA test was used to compare continuous parameters and Chi-square test for comparisons between categorical parameters. The Logistic regression model was used to compare the outcome among all groups. Age and disease severity were adjusted, and a *P* value of <0.05 was considered statistically significant. Moreover, Kaplan-Meier plot was used to compare the survival rate among all groups.

## Results

### Patients Characteristics

**Figure 1:**
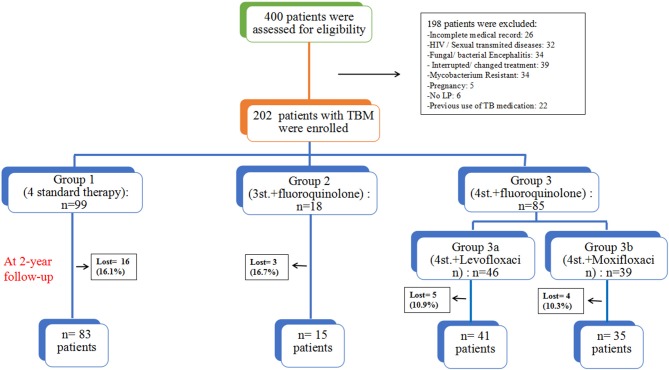
The assessment process and study design; all patients were grouped based on their treatment regimens.

From May 2011 to August 2016, a total of 400 patients with TBM were screened to eligibility, 198 were excluded for different reasons, and only 202 HIV negative patients were enrolled in this study and followed-up for at least 2 years (Figure 1). Based on the published diagnostic criteria, 60 patients (29.7%) had definite TBM, 53 patients (26.2%) had probable TBM and 89 patients (44.1%) had possible TBM. Table 1 shows the baseline characteristics of enrolled patients. There were 115 (56.9%) males and 87 (43.1%) females, their age ranged from 18 to 88 years (median age = 42). There was no significant difference among all groups in terms of gender. Eighty four patients (41.6%) presented with only ≤ 15 days of symptoms; while 89 patients (44.1%) had over 30 days of symptoms at time of admission. The median duration of symptoms before admission was 20 days (range 7–120). At admission there were 74 patients (36.6%) in stage I, 102 (50.5%) were in stage II, and 26 (12.9%) were in stage III. And only 6 patients underwent mechanical ventilation and admitted to ICU, including 4 patients in G1, 1 in G3a, and 1 in G3b. The most common presenting symptoms were headache 194 (96.0%), fever 162 (80.2%), vomiting 120 (59.7%), neck stiffness 104 (51.5%), malaise 96 (47.5%); other less common symptoms included weight loss 48 (23.8%), respiratory distress 34 (16.9%), reduced consciousness 17 (8.4%), convulsion 17 (8.4%), cranial nerve palsy 11 (5.4%), and motor deficit 7 (3.5%). There was no statistical difference among all groups.

**Table 1:** Illustrates the patients' characteristics, lab, and radiological findings.

**Characteristics**	**4-standard therapy *N* = 99**	**3-st.+Levofloxacin *N* = 18**	**4-st.+Levofloxacin *N* = 46**	**4-st.+Moxifloxacin *N* = 39**	**All Patients *N* = 202**	***P*-value**
No. of patients	99/202 (49%)	18/202 (8.9%)	46/202 (22.8%)	39/202 (19.3%)	202 (100%)	
Male sex	57 (57.6%)	9 (50%)	24 (52.2%)	25 (64.1%)	115 (56.9%)	0.657
Age (years), median	41 (16–78)	56 (29–88)	43 (23–77)	35 (18–66)	42 (16–88)	<0.000
body weight (Kg), mean	59.9	50	62.58	56.60	59.50	0.464
Pre–hospital delay (daydays), mean	26.18 ± 23.212	31.39 ± 32.277	29.30 ± 24.574	35.39 ± 30.564	29.12 ± 25.964	0.308
Headache	96 (97%)	17 (94.4%)	42 (91.3%)	39 (100.0%)	194 (96%)	0.198
Fever	75 (75.8%)	14 (77.8%)	39 (84.8%)	34 (87.2%)	162 (80.2%)	0.376
Vomiting	61 (62.2%)	6 (33.3%)	31 (67.4%)	22 (56.4%)	120 (59.7%)	0.080
Neck stiffness	52 (52.5%)	8 (44.4%)	25 (54.3%)	19 (48.7%)	104 (51.5%)	0.880
Hospitalization time (daydays), mean	14.72 ± 9.009	18.50 ± 8.966	20.80 ± 11.923	18.24 ± 11.422	17.20 ± 10.472	<0.012
TB meningitis (grade):						0.138
1	37 (37.4%)	7 (38.9%)	12 (26.1%)	18 (46.2%)	74 (36.6%)	
2	45 (45.5%)	11 (61.1%)	27 (58.7%)	19 (48.7%)	102 (50.5%)	
3	17 (17.2%)	0 (0.0%)	7 (15.2%)	2 (5.1%)	26 (12.9%)	
Neurologic deficit	22 (22.2%)	2 (11.1%)	12 (26.1%)	5 (12.8%)	41 (20.3%)	0.325
**CSF Findings**						
Pleocytosis ≥10 cells/μL	92 (92.9%)	15 (83.3%)	43 (93.5%)	33 (84.6%)	183 (90.6%)	0.280
Low glucose level <50%	79 (79.8%)	10 (55.6%)	38 (82.6%)	30 (76.9%)	157 (77.7%)	0.111
High protein level ≥0.5 g/l	88 (90.7%)	16 (89.9%)	39 (84.8%)	34 (87.2%)	177 (88.5%)	0.762
Pressure mm H_2_O	191.47	181.87	202.68	192.78	193.61	0.798
**Radiology Findings**						
Positive chest CT	56 (56.6%)	5 (27.8%)	28 (60.9%)	29 (74.4%)	118 (58.4%)	<0.010
Hydrocephalus	17 (17.2%)	1 (5.6%)	3 (6.5%)	3 (7.7%)	24 (11.9%)	0.154
Meningeal enhancement	31 (31.3%)	6 (35.3%)	9 (19.6%)	16 (41.0%)	62 (30.8%)	0.187
Tuberculoma	12 (12.1%)	3 (16.7%)	10 (21.7%)	11 (28.2%)	36 (17.8%)	0.166
Infarction	22 (22.2%)	4 (22.2%)	9 (19.6%)	5 (12.8%)	40 (19.8%)	0.653
Basal heyperintensity	2 (2.0%)	1 (5.6%)	5 (10.9%)	3 (7.7%)	11 (5.4%)	0.153
**Mycobacterium Testing**						
T-spot (+)	18 (18.2%)	3 (16.7%)	13 (28.3%)	21 (53.8%)	55 (27.2%)	<0.000
TB-DNA (+)	14 (14.1%)	2 (11.1%)	14 (30.4%)	10 (25.6%)	40 (19.8%)	0.073
Anti-TB Ab (+)	11 (11.1%)	2 (11.1%)	7 (15.2%)	7 (17.9%)	27 (13.4%)	0.716
TB culture (+)	1 (1.0%)	0 (0.0%)	2 (4.3%)	1 (2.6%)	4 (2%)	0.86
PPD	15 (15.2%)	2 (11.1%)	4 (8.7%)	7 (17.9%)	28 (13.9%)	0.609

### Laboratory Data and Radiological Findings

The biochemical and radiological findings are shown in Table 1. Cerebrospinal fluid (CSF) analysis showed pleocytosis with a predominance of lymphocytes in 183 (90.6%) patients, a low glucose level in 157 (77.7%), and raised protein concentration in 177 (88.5%), which is consistent with the diagnosis of TBM. Except for Interferon-gamma release assays (IGRAs), which was higher in groub3b; there was no statistical difference among all groups. IGRAs was positive in 55 patients (27.2%); TB-DNA was found in 40 patients (19.8%); anti-TB antibody was detected in 27 patients (13.4%); TB culture was positive in 4 (2.0%); and the purified protein derivative (PPD) test was positive in 28 (13.9%). Neuroradiological findings included leptomeningeal enhancement in 62 patients (30.8%); infarction in 40 (19.8%); tuberculoma in 36 (17.8%); hydrocephalus in 24 (11.9%); pre-contrast basal hyperintensity in 11 (5.4%); and positive chest CT suggested pulmonary tuberculosis in 118 (58.4%). Except for positive chest CT, which was lower in groub2, there was no statistical difference among all groups.

### Outcome

The overall outcome was compared at different time points (1, 1.5, and 2 years). At 2 years follow-up, 28 (13.9%) patients were lost, including 16 (16.2%) patients in *Group1* (4st. Regimen); 3 (16.7%) in *Group2* (3st.+L regimen); 5 (10.9%) in *group3a* (4st.+Levofloxacin regimen); and 4 (10.3%) in *group3b* (4st.+Moxifloxacin regimen), there was no significant difference among all groups. All those who dropped were considered to have a poor outcome (death) during the analysis of the results. This study found that the 4-drug standard therapy was not inferior to other intensified therapies with fluoroquinolones for HIV-negative patients with TBM. Moreover, Hydrocephalus was the only independent risk factor for a poor outcome in these patients.

**Figure 2:**
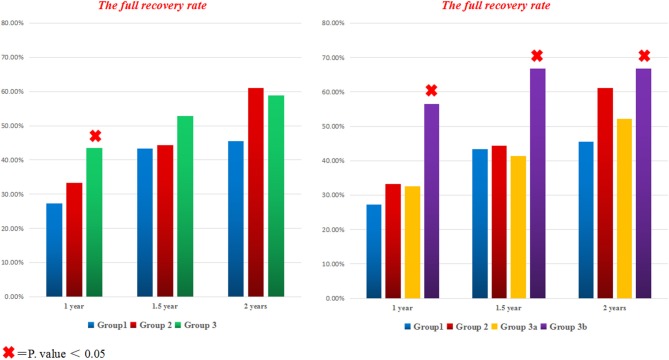
The full recovery rate for all groups at different time points. The bar graph on the left side compares the percentage of fully recovered patients with TBM in all different treatment groups; while the bar graph on the right side contrasts the full recovery rate in all groups after subdividing group3 to A (with levofloxacin) and B (with Moxifloxacin). × = *P* < 0.05.

**Figure 3:**
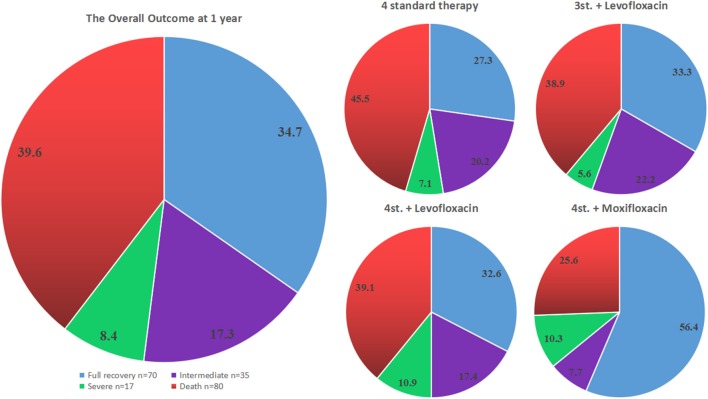
The outcome of all different groups at 12 months from admission. The pie chart on the left side represents the overall outcome for all patients, while the 4 pie charts on the right side show the outcome for each treatment group separately.

At 1 year follow up, the full recovery rate in *Group3* was statistically significant compared to standard therapy (OR = 2.056; 95% CI: 1.110–3.806; *P* = 0.022), even after adjustment for both age and disease severity (adjusted OR = 2.015; 95% CI: 1.079–3.764; *P* = 0.028). Surprisingly, further subdivision of *group3* based on the used fluoroquinolone agent revealed that *group3a* was not statistically different from *groups 1* and 2 (adjusted OR = 1.334; 95% CI: 0.619–2.875; *P* = 0.461); while the full recovery rate in *group3b* was significantly superior to other treatment regimens (adjusted OR = 3.201; 95% CI: 1.456–7.038; *P* = 0.004) (Figures 2, 3). Furthermore, *Group3b* regimen remained significantly better than other treatment regimens over the following year, with the adjusted OR = 2.598 (95% CI: 1.172–5.762; *P* = 0.019) at 1.5 year and 2.361 (95% CI: 1.069–5.215; *P* = 0.034) at 2 years (Table 2).

**Table 2:** Shows the outcome and complications for all groups.

**OUTCOME**	**4-standard therapy *N* = 99**	**3-st.+Levofloxacin *N* = 18**	**4-st.+Levofloxacin *N* = 46**	**4-st.+Moxifloxacin *N* = 39**	**All Patients *N* = 202**	***P*–value**
**THE FULL RECOVERY RATE**						
At 1 year	27 (27.3%)	6 (33.3%)	15 (32.6%)	22 (56.4%)	70 (34.7%)	<0.014
At 1.5 year	43 (43.4%)	8 (44.4%)	19 (41.3%)	26 (66.7%)	96 (47.5%)	0.066
At 2 years	45 (45.5%)	11 (61.1%)	24 (52.2%)	26 (66.7%)	106 (52.5%)	0.130
Death rate	44 (44.4%)	8 (44.4%)	18 (39.1%)	10 (25.6%)	80 (39.6%)	0.228
**THE OVERALL GOOD OUTCOME (mRS 0–2)**						
At 1 year	47 (47.5%)	10 (55.6%)	23 (50.0%)	25 (64.1%)	105 (52.0%)	0.352
At 2 years	51 (51.5%)	11 (61.1%)	26 (56.5%)	27 (69.2%)	115 (56.9%)	0.239
Hepatotoxicity	3 (3.0%)	3 (16.4%)	3 (6.5%)	6 (15.4%)	15 (7.4%)	<0.034
Renal impairment	3 (3.0%)	1 (5.6%)	4 (8.7%)	0 (0.0%)	8 (4.0%)	0.198
Hematotoxicity	3 (3.0%)	1 (5.6%)	2 (4.3%)	4 (10.3%)	10 (5.0%)	0.368
Cardiotoxicity	1 (1.0%)	0 (0.0%)	1 (2.2%)	1 (2.6%)	3 (1.5%)	0.826
Hypersesitivity	3 (3.0%)	2 (11.1%)	1 (2.2%)	3 (7.7%)	9 (4.5%)	0.276
Disability	6 (6.1%)	0 (0.0%)	3 (6.5%)	1 (2.6%)	10 (5.0%)	0.591
Visual impairment	3 (3.0%)	1 (5.6%)	1 (2.2%)	1 (2.6%)	6 (3.0%)	0.910
Seizure	11 (11.1%)	0 (0.0%)	3 (6.5%)	2 (5.1%)	16 (7.9%)	0.325

However, the assessment of the overall outcome at 1 year based on mRS classification did not show a statistical significance among all groups (*P* = 0.397). Even a further subdivision of group G3 did not show any significant difference; in particular, the outcome of G3b was not statistically significant compared to other groups (adjusted OR = 1.985; 95% CI: 0.907–4.342; *P* = 0.086) (Table 2; Figure 3).

At 2 years follow up, although the overall outcome of G3b was better than other groups but did not reach a statistical significance (adjusted OR = 2.157; 95% CI: 0.969–4.803; *P* = 0.060) (Figure 6). Moreover, the Hazard ratio (HR) of G3b compared to G1 was 0.53 (95% CI: 0.262–1.058, *P* = 0.072) after adjusting for age, disease severity, while the HR of G3a was 0.88 (95% CI: 0.500–1.563, *P* = 0.672).

The most frequent complications are shown in Table 2, except for hepatotoxicity which was significantly higher in the intensified groups (*P* = 0.034); there was no statistical difference among all groups. It is also worth mentioning that 17 (8.4%) patients had stopped all anti-tuberculosis drugs at a time point during the treatment period, and later some or all the drugs were re-introduced according to the case.

### Predictive Factors of TBM Prognosis

**Figure 4:**
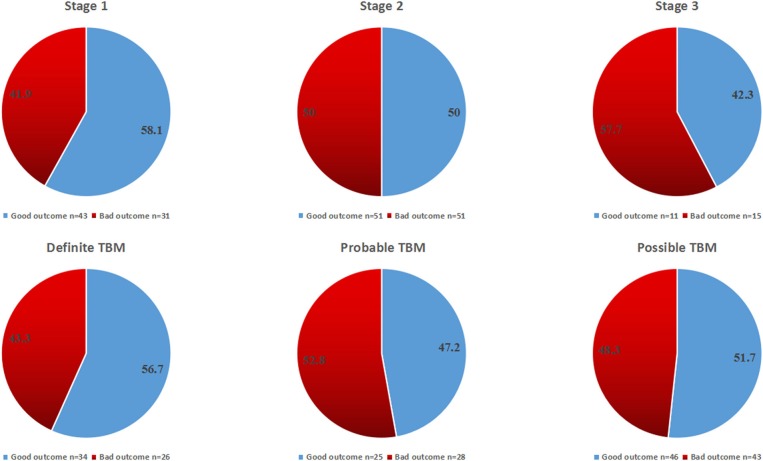
The overall outcome at 12 months for all patients according to disease stages and diagnostic categories.

**Figure 5:**
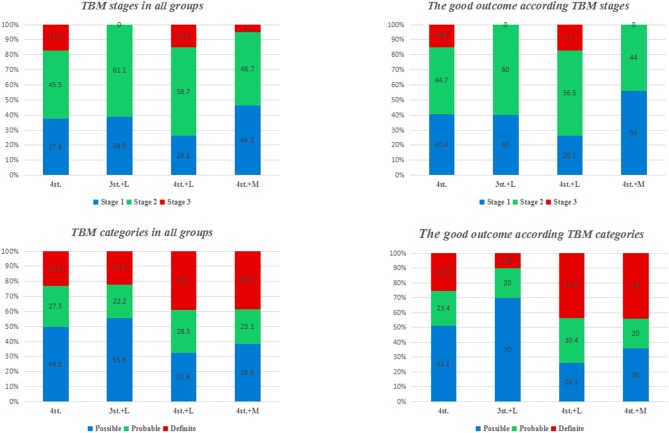
the percentage of TBM patients in each stage and category at admission; and the percentage of patients with good outcome in each stage and category.

The overall outcome at 1 year according to TBM stages and categories is shown in Figures 4, 5. There was no statistical difference among all groups.

Univariate analysis revealed that the factors impacting the outcome of TBM patients at 1 year were Hydrocephalus (OR = 3.759, 95% CI: 1.425–9.918, *P* = 0.007), Tuberculoma (OR = 2.200, 95% CI:1.043–4.637, *P* = 0.038), Respiratory distress (OR = 2.273, 95% CI:1.056–4.892, *P* = 0.036). However, multivariate analysis has shown that hydrocephalus (OR = 3.557, 95% CI: 1.326–9.543, *P* = 0.012) was the only independent risk factor for a poor outcome at 1 year. Whereas, multivariate analysis at 2 years follow-up has shown that hydrocephalus (OR = 3.461, 95% CI: 1.349–8.882, *P* = 0.010) and respiratory distress (OR = 2.290, 95% CI: 1.055–4.973, *P* = 0.036) were independent risk factors for a poor outcome. T-spot was the only favorable prognostic factor at 1 year (OR = 2.142, 95% CI: 1.126–4.075, *P* = 0.020) (Table 3).

**Table 3:** Univariable and multivariable logistic regression analyses.

**Variable**	**Odd ratio**	**95% CI**	***P* value**
**UNIVARIABLE LOGESTIC REGRESSION ANALYSIS**			
Age	1.565	0.716–3.422	0.262
Male sex	1.359	0.776–2.378	0.283
Admission delay >15 daydays	1.169	0.666–2.054	0.586
Headache	8.089	0.977–67.00	0.053
Fever	1.026	0.513–2.052	0.941
Vomiting	1.359	0.771–2.395	0.289
Neck stiffness	1.497	0.859–2.608	0.155
Convulsion	1.043	0.386–2.821	0.934
Nerve palsy	1.661	0.471–5.859	0.430
Neurological deficit	1.231	0.618–2.454	0.555
Advanced disease stage	1.447	0.829–2.630	0.186
Respiratory distress	2.273	1.056–4.892	0.036^*^
Positive chest x–ray	1.211	0.691–2.122	0.505
Hydrocephalus	3.759	1.425–9.918	0.007^*^
Meningeal enhancement	1.250	0.686–2.276	0.466
Tuberculoma	2.200	1.043–4.637	0.038^*^
Infarction	1.951	0.951–4.005	0.068
Basal hyperintensity	1.250	0.686–2.276	0.466
Pleocytosis	1.976	0.744–5.247	0.171
Low glucose level	1.519	0.775–2.979	0.224
High protein level	1.472	0.613–3.534	0.387
T–spot	2.142	1.126–4.075	0.020^*^
TB–DNA	1.163	0.581–2.331	0.670
Anti–TB Ab	1.181	0.523–2.667	0.690
PPD	1.096	0.493–2.436	0.821
**MULTI–VARIABLE LOGISTIC REGRESSION ANALYSIS**			
Hydrocephalus	3.557	1.326–9.543	0.012^*^
Tuberculoma	2.027	0.937–4.384	0.073
Respiratory distress	1.885	0.849–4.186	0.119

* *P < 0.05*.

## Discussion

In fact, to date, the optimal treatment regimen for TBM is still unclear. However, in recent years, considerable studies have proposed few drugs of the fluoroquinolone class as viable and good alternatives to existing anti-TB drugs (6–9). In general, the fluoroquinolones have potent bactericidal activity against *M. tuberculosis*, with Moxifloxacin being more potent than others (11, 13–17).

In terms of full recovery, our study has shown that only the intensified regimen with Moxifloxacin given for 2 months was associated with a significantly better outcome at all-time points of this study. Particularly patients taking 4st.+Moxifloxacin had 3.2 more chances to achieve a full recovery at 1 year, 2.6 chances at 1.5 years and 2.4 chances at 2 years follow-up relative to those on 4 drugs standard therapy alone. However, the assessment of the overall outcome using mRS classification did not show any statistical difference among all groups at 1 year (*P* = 0.397) and 2 years (*P* = 0.295) follow up. This is certainly because the good outcome group included the fully recovered patients (mRS 0) and patients with mild symptoms or minor disability (mRS 1–2) (Figure 3). Although the addition of Moxifloxacin, but not Levofloxacin, may enhance the effect of Mycobacterium killing and lead to a better outcome compared to the standard and other intensified regimens, it is still not sufficient enough to conclude the superiority of 4 standard therapy plus Moxifloxacin over other regimens just based on a single retrospective study. Therefore, a well-designed prospective study with bigger sample size is needed to confirm this result.

Though many publications regarding TBM management are available; most of these studies were conducted in different centers under different conditions, which resulted in obviously controversial findings. Therefore, it is difficult to compare the outcome of these studies and to apply their findings in clinical practice. To our knowledge, this is the first conducted study comparing the efficacy of 4-drug standard therapy and 3 different intensified therapies with different types of fluoroquinolones in one center. It is also worth mentioning that the treatment selection was based on the physician's experience and preference.

A recent clinical trial of 817 adults with TBM compared standard therapy plus Levofloxacin, with standard therapy alone. Surprisingly, the intensified regimen was not associated with improved survival compared to the standard regimen over 9 months of follow-up with 28% mortality in both arms (12). In our study, the two intensified treatment regimens with Levofloxacin (4-standard therapy +Levofloxacin; 3-standard regimen + Levofloxacin) did not show any advantages over the standard regimen at all-time points (*P*-value at 1 year was 0.462 and 0.621, respectively; while at 1.5 years was 0.869 and 0.760, respectively; and at 2 years was 0.430 and 0.411, respectively) (Figure 2), this finding is consistent with the recently published literature (12).

In the Indonesian trail, the mortality rate at 6 months follow up had reduced from 65 to 34% in patients receiving high dose IV rifampicin for the initial 2 weeks; in this study, moxifloxacin did not appear to have a significant impact on mortality (18). In contrast, our study has shown that 4-standard therapy plus Moxifloxacin was the only intensified regimen that associated with a statistically significant better outcome (full recovery rate) at all-time points (at 1 year *P* = 0.002; 1.5 year *P* = 0.016; 2 years *P* = 0.027) (Figure 2). The possible explanation for our finding might be because our patients had used a standard dose of Moxifloxacin for 8 weeks (long enough to enhance bacterial killing over a long period), but the Indonesian patients had used it for only 2 weeks. Besides, the endpoint time for the Indonesian study was just 6 months; instead, our patients were evaluated for a longer period, up to 2 years. Therefore, we propose that drug exposure time and the endpoint time may have a profound impact on the overall outcome in the two studies. In addition, Nijland et al. and Alffenaar et al. had observed that Co-administration of rifampicin with moxifloxacin reduces the plasma concentration of moxifloxacin in patients with TB (11, 19), which might even have shortened the exposure time of moxifloxacin in the Indonesian patients. In contrast, our patients had a longer administration period that may have played an essential role in building up an effective concentration of moxifloxacin in their bodies, which ultimately led to a better outcome.

The previous studies have reported numerous risk factors that may impact the outcome of TBM. Advanced age, altered consciousness, positive TB culture, immunosuppression, hydrocephalus, tuberculoma, neurological deficits have all been reported to predict unfavorable outcome (20–24). However, in recent years, these predictors might have changed with the advances in diagnosis and treatment. More recent studies have reported that old age and hydrocephalus were essential predictors of poor prognosis (25, 26). In the current study hydrocephalus was the only independent risk factor for poor prognosis at 1 and 2 years (Table 3).

**Figure 6:**
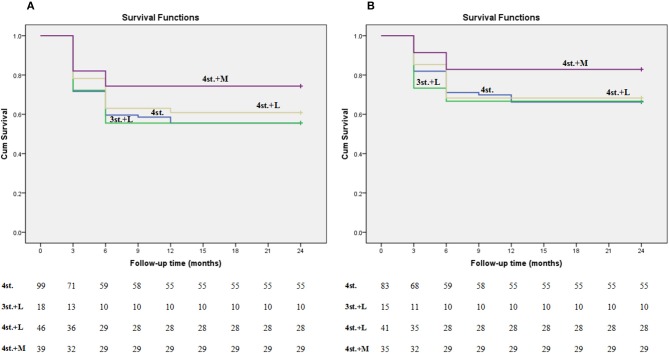
Kaplan-meier plot shows the survival rate among all groups at 2 years follow up. Plot **(A)** represents all patients including the lost ones; while plot **(B)** represents only the patients who finished the study (the lost patients were excluded).

In our study, 63.4% of the patients presented with advanced clinical features (Figure 5; Table 1). Several studies had reported similar observations (27, 28). TBM mortality rate ranges from 6 to 65%, with higher rates been reported in HIV co-infected individuals (29). The overall mortality rate at 2 years in our study was 39.6%; this is might be due to the exclusion of patients with multidrug-resistant TBM and/ or sexually transmitted diseases which are major prognostic factors for patients with TBM (30); moreover, around 40% of our patients were diagnosed and treated before the onset of coma (Figure 6).

The limitations of our study included being a retrospective observational study, lack of randomization, and prone to selection bias; the contact history of many patients was not clear so that we couldn't analyze it; the study sample was small, especially in the 2nd regimen with Levofloxacin group. Therefore, all these factors and others may have potentially influenced the results. Further prospective studies with bigger sample size are required to clarify this finding.

## Conclusion

In our study, the overall outcome in patients with TBM at 1 and 2 years follow up did not show any statistical difference between the 4-standard chemotherapy and other intensified treatment regimens. Moreover, Hydrocephalus was the only independent risk factor for poor prognosis at 1 and 2 years.

## Ethics Statement

The study's objectives and procedures were approved by the Ethics Committee of West China Hospital of Sichuan University. No written consent was given by the participants because the data were analyzed anonymously.

## Author Contributions

AA made substantial contributions to conception and design of study, drafted and revised the manuscript, read and approved the final manuscript. YR and WL made data collection, analysis, and interpretation of data, read and approved the final manuscript. JL drafted and revised the manuscript, read and approved the final manuscript. DZ made substantial contributions to conception and design of study, revised the manuscript, read and approved the final manuscript.

### Conflict of Interest Statement

The authors declare that the research was conducted in the absence of any commercial or financial relationships that could be construed as a potential conflict of interest.

## Abbreviations

TBM: Tuberculous meningitis
TB: Tuberculosis
WHO: World Health Organization
CSF: cerebrospinal fluid
BMC: British Medical Research Council
mRS: modified Rankin Scale
IGRAs: Interferon-gamma release assays
PPD: purified protein derivative
CT: computed tomography.
